# Spatial transcriptome atlas reveals pulmonary microstructure-specific COVID-19 gene signatures in cynomolgus macaques

**DOI:** 10.1038/s42003-023-05253-8

**Published:** 2023-08-28

**Authors:** Taehwan Oh, Green Kim, Seung Ho Baek, YoungMin Woo, Bon-Sang Koo, Eun-Ha Hwang, Kyuyoung Shim, You Jung An, Yujin Kim, Jinyoung Won, Youngjeon Lee, Kyung Seob Lim, Jae-Hak Park, Jung Joo Hong

**Affiliations:** 1 National Primate Research Centre, Korea Research Institute of Bioscience and Biotechnology (KRIBB), Cheongju, Chungcheongbuk Republic of Korea; 2 KRIBB School of Bioscience, Korea University of Science & Technology (UST), Daejeon, Republic of Korea; 3 Futuristic Animal Resource and Research Center, Korea Research Institute of Bioscience and Biotechnology (KRIBB), Cheongju, Republic of Korea; 4 Department of Laboratory Animal Medicine, College of Veterinary Medicine, Seoul National University, Seoul, Republic of Korea

**Keywords:** Pathogenesis, Infection

## Abstract

Characterizing the host response to severe acute respiratory syndrome coronavirus 2 (SARS-CoV-2) at the molecular level is necessary to understand viral pathogenesis and identify clinically relevant biomarkers. However, in humans, the pulmonary host response during disease onset remains poorly understood. Herein, we utilized a spatial transcriptome atlas to identify pulmonary microstructure-specific COVID-19 gene signatures during the acute phase of lung infection in cynomolgus macaques. The innate immune response to virus-induced cell death was primarily active in the alveolar regions involving activated macrophage infiltration. Inflamed vascular regions exhibited prominent upregulation of interferon and complement pathway genes that mediate antiviral activity and tissue damage response. Furthermore, known biomarker genes were significantly expressed in specific microstructures, and some of them were universally expressed across all microstructures. These findings underscore the importance of identifying key drivers of disease progression and clinically applicable biomarkers by focusing on pulmonary microstructures appearing during SARS-CoV-2 infection.

## Abstract (web summary)

A spatial transcriptome atlas identifies pulmonary microstructure-specific COVID-19 gene signatures during the acute phase of lung infection in cynomolgus macaques.

## Introduction

Severe acute respiratory syndrome virus (SARS-CoV-2), the virus causing coronavirus disease 2019 (COVID-19), has fueled a global pandemic during the past few years^1,2^. Although the development of effective vaccines has reduced the threat of COVID-19 to endemic regions, the emergence of new variants and the possibility of reinfection in recovered patients continue to pose a threat to public health. The disease spectrum of COVID-19 varies from asymptomatic infection to acute respiratory distress syndrome (ARDS), which is often accompanied by immune dysregulation and a “cytokine storm”^3^. In particular, patients with ARDS exhibit diffuse alveolar damage, which results in pulmonary edema and respiratory insufficiency. Severe cases also exhibit vascular endothelialitis and thrombosis^4^. Moreover, patients with SARS-CoV-2 infection may exhibit a complex disease progression, with varying patterns of pathological changes in the epithelial and vascular tissues, occasionally occurring as a single pattern or both patterns simultaneously^5^. Therefore, a comprehensive histopathological assessment is required to fully understand and manage this disease.

Defining of a host response to SARS-CoV-2 at the molecular level is necessary to understand viral pathogenesis and identify clinical biomarkers. Using single-cell or bulk RNA sequencing, it is possible to predict prognosis in mild and severe patients and to screen individuals at a high risk for infection^6,7^. However, these results were obtained from nasal swab and blood sample testing; therefore, it is unclear whether virus-induced tissue damage—the target of clinical treatment—is accurately reflected using such methods. Notably, recent advances in spatial transcriptomics have enabled studies on host response to a virus at single-cell resolution without losing topological information. The spatially resolved transcriptome of human lungs infected with SARS-CoV-2 has revealed that the virus infects alveolar epithelial cells and induces localized hyperinflammation at the site of infection^8,9^. In a previous study, intra-pulmonary heterogeneity of the host response to SARS-CoV-2 infection in humans was identified, particularly with respect to the interferon response, which is dependent on virus distribution^10^. Moreover, tissue-type-specific responses to SARS-CoV-2 were identified, with prominent expression of genes associated with macrophage activation, cytokine pathways, and complement activation in alveoli, large airways, and blood vessels, respectively^11^.

Previous attempts to elucidate viral pathogenesis using spatial transcriptome analysis were done with samples from deceased patients, who had succumbed to severe infection. However, because autopsy samples are obtained at the terminal stage of the disease, understanding the mechanism of disease progression at earlier stages of infection is limited. Moreover, because SARS-CoV-2 infection has many confounding factors, including pre-existing host immunity^12,13^, caution is necessary when interpreting the results of clinical studies. Nonhuman primates (NHPs), which are phylogenetically the closest animals to humans, are widely used as animal models to evaluate the efficacy of vaccine candidates against SARS-CoV-2 or identify the pathogenicity of emerging variants of concern. Such models are essential to support research on human patients, because not only can they establish the route and amount of virus infection, but also provide an opportunity for longitudinal sampling according to the research purpose. Therefore, spatial transcriptome studies using a sophisticated NHP model are needed to better understand the underlying mechanism of SARS-CoV-2 infection.

In this study, we present a whole transcriptome atlas of SARS-CoV-2-infected cynomolgus macaque lungs obtained by Nanostring GeoMX technology. The spatially resolved transcriptomic profiles from lung tissues confirmed the expression of the host response at the site of virus infection, and this response was directly associated with tissue damage. The objective of the study was to elucidate the pathogenesis of early SARS-CoV-2 infection by classifying the expression patterns of the host response to the virus according to the microstructure of the lung.

## Results

### Histopathological characterization of SARS-CoV-2 infected lungs

Histopathological examinations were conducted on the lungs of SARS-CoV-2 and mock-infected cynomolgus macaques. These lungs were divided into six lobes (left upper, left middle, left lower, right upper, right middle, and right lower lobes) and pathological changes in the alveoli, bronchioles, and blood vessels were assessed for each lobe. In the virus-infected lung lobes, diffuse alveolar damage was a typical lesion, which was characterized by alveolar wall thickening, epithelial damage, and proteinosis. Various inflammatory cells, including neutrophils, lymphocytes, and macrophages were found to be infiltrated in the damaged alveoli. Furthermore, pulmonary edema and hyaline membrane formation were observed in severely damaged lung lobes. Bronchiolar epithelial degeneration was concurrently observed with mononuclear cell infiltration. Neutrophils tended to infiltrate less than mononuclear cells, but were often present with exudates in severely damaged bronchioles. Peribronchiolar inflammatory cuffing was observed multifocally, but was not observed in several lung lobes. Vascular endothelial damage was observed concurrently with intravascular neutrophil and mononuclear cell infiltration, and was often accompanied by perivascular inflammation (Table 1). These results indicate that SARS-CoV-2 induces acute lesions in the alveolar, bronchiolar, and vascular regions of cynomolgus macaque lungs, indicating the severe COVID-19 infection.

**Table 1:** Pulmonary histopathological findings in cynomolgus macaques infected with SARS-CoV-2.

	Macaque #1						Macaque #2						Macaque #3					
Lesions	LU	LM	LL	RU	RM	RL	LU	LM	LL	RU	RM	RL	LU	LM	LL	RU	RM	RL
Alveolar wall thickening	2	2	2	1	2	2	1	1	1	2	2	2	2	1	2	1	1	1
Alveolar epithelial damage	0	2	0	3	1	0	1	0	3	1	0	0	0	0	1	1	0	0
Alveolar hemorrhage	0	1	0	0	0	0	0	0	0	3	3	3	0	0	0	0	0	0
Capillary congestion	1	1	1	0	1	1	0	0	0	1	1	1	1	1	1	1	0	0
Alveolar proteinosis	3	3	3	3	3	3	1	1	3	3	3	3	3	1	3	1	1	1
Pulmonary edema	2	2	2	3	2	2	2	2	3	2	2	2	2	2	3	2	0	0
Hyaline membrane	0	3	0	3	0	0	1	1	3	0	0	0	0	0	1	0	0	0
Intra-alveolar neutrophil	2	2	2	3	2	2	1	1	3	2	2	2	2	1	2	1	0	1
Intra-alveolar lymphocyte	2	2	2	1	2	2	1	1	1	1	2	2	2	1	2	1	1	1
Intra-alveolar macrophage	2	3	2	3	2	2	0	0	3	1	2	2	2	1	2	1	0	0
Bronchiolar epithelial degeneration	2	2	3	2	2	N/A	1	2	2	1	1	3	2	N/A	1	N/A	3	2
Intra-bronchiolar neutrophil	0	2	3	1	2	N/A	0	1	2	1	1	2	1	N/A	1	N/A	3	1
Intra-bronchiolar mononuclear cell	1	3	3	1	2	N/A	1	2	2	1	1	2	1	N/A	1	N/A	3	2
Peribronchiolar inflammatory cuffing	0	1	1	1	1	N/A	0	1	2	1	1	2	0	N/A	1	N/A	0	2
Vascular endothelial damage	1	2	0	3	2	2	2	2	3	1	1	1	1	1	2	1	0	0
Intra-vascular neutrophil	1	2	1	3	1	1	1	1	3	1	1	1	1	1	3	2	0	0
Intra-vascular mononuclear cell	1	2	1	3	1	1	1	1	3	1	1	1	1	1	1	2	0	0
Perivascular inflammation	1	1	1	1	1	1	2	2	1	1	1	1	1	1	2	1	0	0

Histopathological changes were scored from 0 to 4: absent (0); none of the slide affected, minimal (1); <10% of slide affected, mild (2); 10–30% of slide affected, moderate (3); 30–50% of slide affected, and severe (4); >50% of slide affected. N/A indicates not applicable.

*LU* left up, *LM* left mid, *LL* left low, *RU* right up, *RM* right mid, *RL* right low.

### Region of interest (ROI) selection for digital spatial profiling

**Fig. 1:**
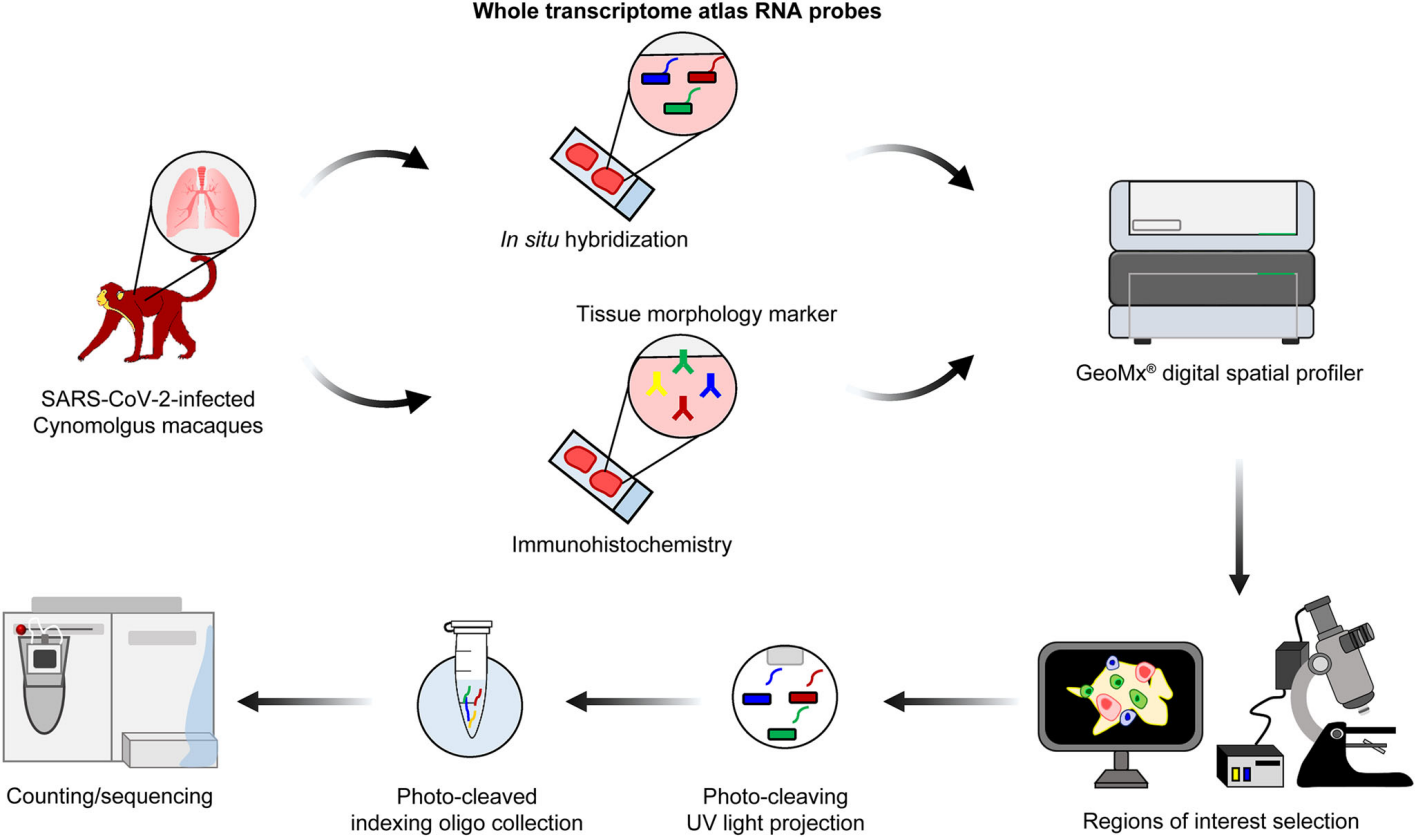
Schematic design of digital spatial profiling for whole transcriptome analysis. SARS-CoV-2-infected tissue sections were prepared and immunohistochemistry was performed using the tissue morphology markers. The tissue slides were then hybridized with probes from the Human Whole Transcriptome Atlas. Subsequently, they were loaded onto the GeoMx digital spatial profiler. After selection of the region of interest (ROI), oligos from the hybridized probes were released by photo-cleaving UV light, and deposited into a 96-well plate. Finally, sequencing libraries were constructed and subsequent sequencing and counting were carried out.

**Fig. 2:**
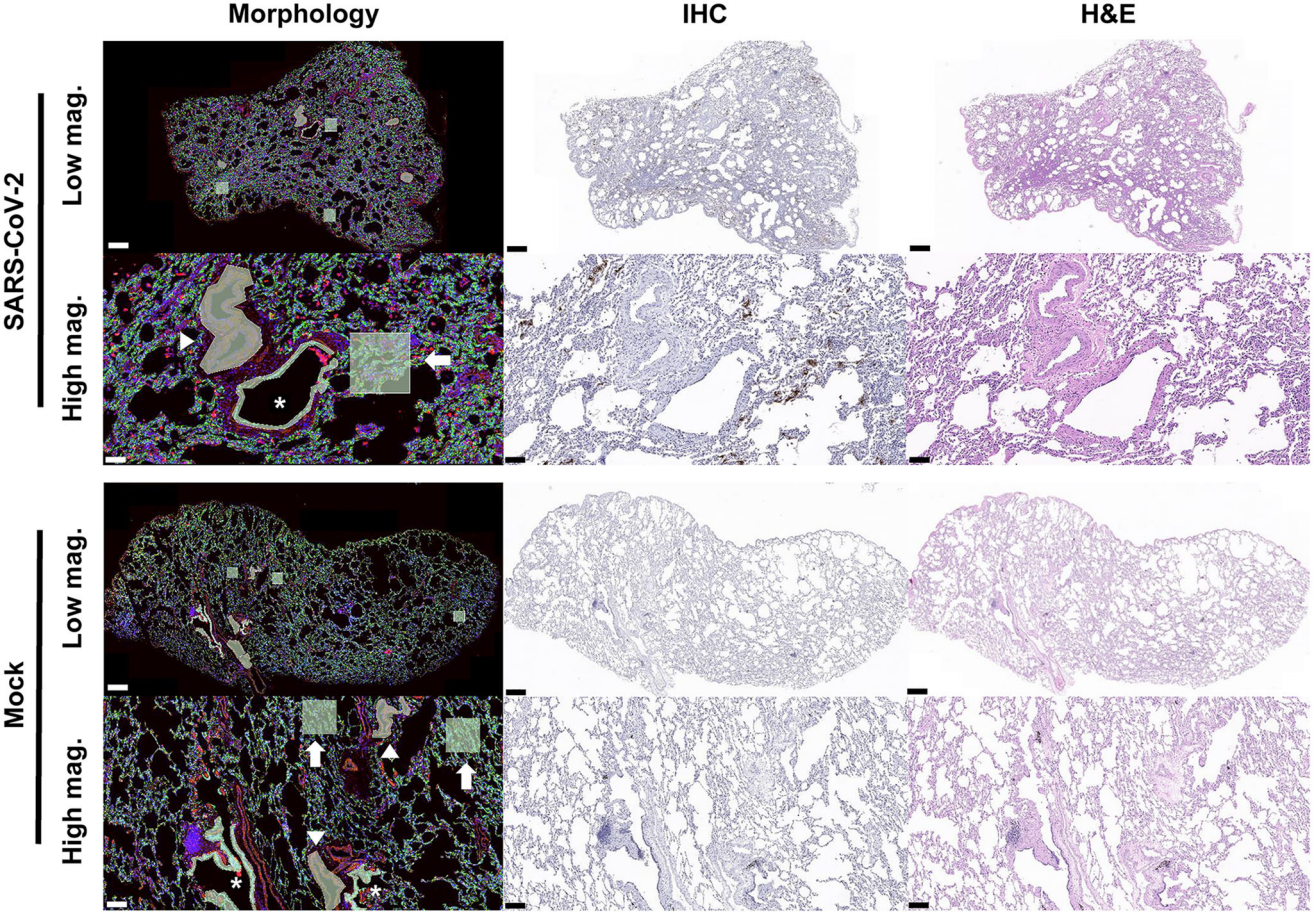
Immunohistochemistry and H&E staining for digital spatial profiling. Tissue morphology was visualized with the epithelial cell marker PanCK (green), immune cell marker CD45 (yellow), and macrophage marker CD68 (red) along with Syto13 DNA nucleic acid (blue) staining. Tissue microstructures were annotated as arrow; alveolar, asterisk; bronchiolar, and arrowhead; vascular ROIs. IHC indicates DAB-labeling (brown) immunohistochemistry for SARS-CoV-2 viral nucleic acid. H&E indicates haematoxylin and eosin staining. Scale bar, SARS-CoV-2 low magnification; 500 μm and high magnification; 100 μm. Scale bar, Mock low magnification; 500 μm and high magnification; 200 μm.

NanoString GeoMx was used to conduct a spatial transcriptomic analysis of lung tissues obtained from experimentally infected animals (Fig. 1). Spatial transcriptional profiling of the target ROI was guided by the distribution of SARS-CoV-2 nucleocapsid. Virus-infected lung lobes exhibited a strong signal for viral nucleocapsid, whereas mock-infected lung lobes exhibited no IHC signal. The positive signals were primarily detected within type 2 pneumocytes and macrophages and occasionally within bronchiolar epithelial cells. Further, the ROIs were selected on serial sections visualized with the epithelial cell marker PanCK, immune cell marker CD45, and macrophage marker CD68 along with Syto13 DNA nucleic acid staining. The target ROIs were then classified into three major pulmonary structures: alveolar, bronchiolar, and vascular ROIs. Overall, 12 ROIs (4 for each structural ROI) were selected for each macaque. In total, 36 ROIs were selected from virus-infected macaques. Similarly, 36 ROIs were selected in the lung lobes of mock-infected macaques exhibiting normal morphology (Fig. 2).

### Dimensionality reduction in the spatial transcriptomic dataset

**Fig. 3:**
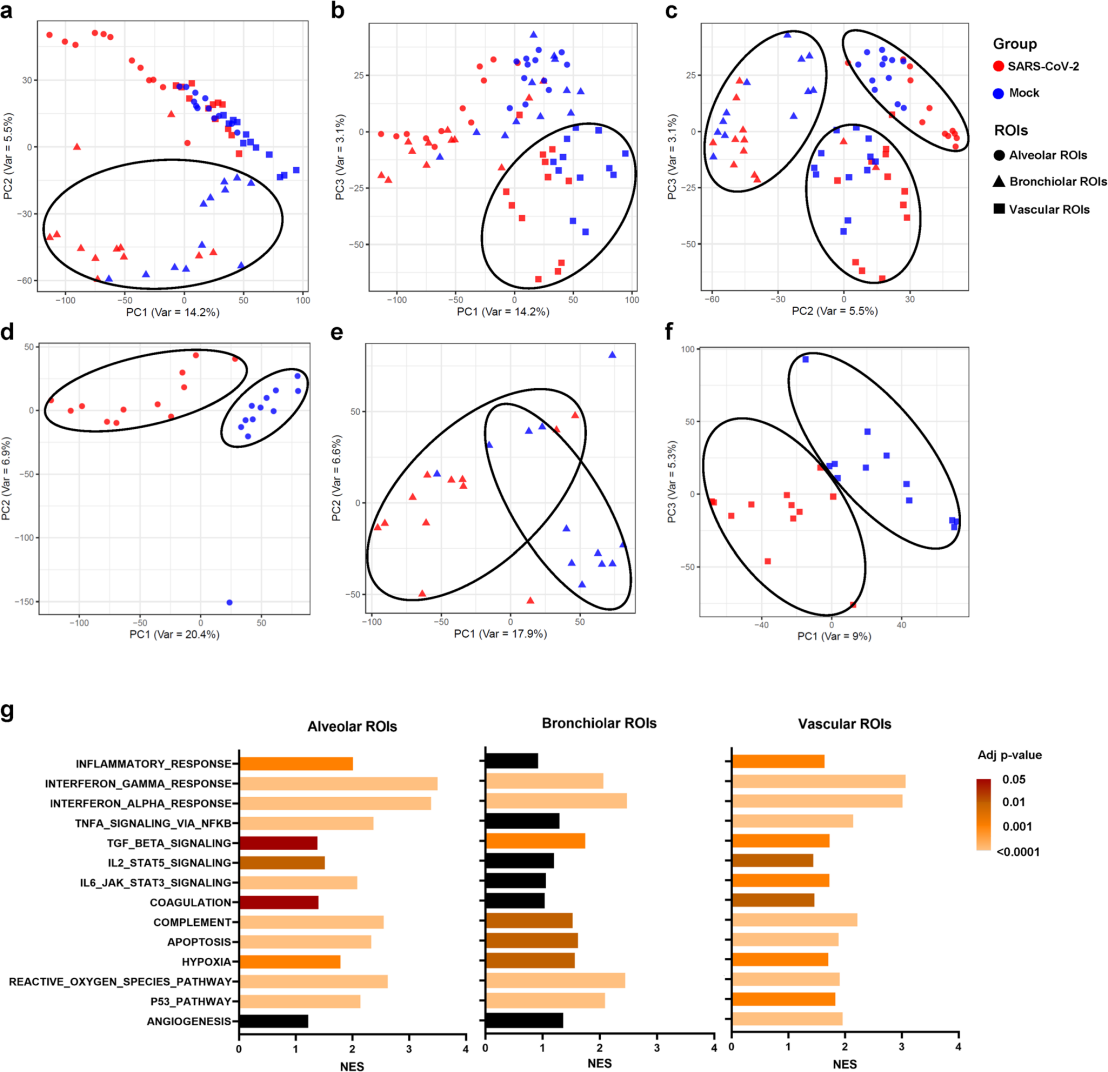
Principal component analysis (PCA) and gene set enrichment analysis of the spatial transcriptome dataset. PCA graphs represent all (**a**–**c**), alveolar (**d**), bronchiolar (**e**), and vascular (**f**) ROIs selected from SARS-CoV-2 and mock-infected lungs. Each group and microstructural ROI are represented using a different color and marker, respectively. The normalized enrichment score (NES) is presented as a bar graph and adjusted p-values are represented by a color gradient (**g**). The black color indicates non-significance.

Principal component analysis (PCA) was used as a data dimensionality reduction method to identify biological factors causing variability in the spatial transcriptome. When all ROIs were collectively analyzed, PC1 and PC2 distinguished bronchiolar ROIs from other structural ROIs (Fig. 3a). In addition, vascular ROIs were separated from other structural ROIs when plotting PC1 and PC3 (Fig. 3b). The plots of PC2 and PC3 could cluster alveolar, bronchiolar, and vascular ROIs (Fig. 3c). Notably, a plot of alveolar ROIs on PC1 and PC2 revealed that SARS-CoV-2-infected ROIs were clearly separated from mock-infected ROIs (Fig. 3d). In the PCA plot of bronchiolar ROIs, several mock-infected ROIs overlapped with SARS-CoV-2-infected ROIs in PC1 and PC2 (Fig. 3e). Vascular ROIs were clustered by SARS-CoV-2-infected ROIs and mock-infected ROIs in plots of PC1 and PC3 (Fig. 3f). These results indicate that structural heterogeneity with the dominant cell type is associated with variability in the spatial transcriptome and SARS-CoV-2 infection-induced transcriptional alterations in each targeted structural ROI.

### Spatial transcriptomic profile of the pulmonary host response to SARS-CoV-2

We conducted GSEA to identify the underlying biological process associated with each structural ROI by confirming the enrichment or depletion pattern of gene sets grouped by their functional pathways. First, in alveolar ROI, we found a significant enrichment of gene sets, including the inflammatory response, interferon gamma response, interferon alpha response, TNF-alpha signaling through NFkB, TGF beta signaling, IL2–STAT5 signaling, IL6–JAK–STAT3 signaling, complement, apoptosis, hypoxia, reactive oxygen species pathway, and p53 pathway. Second, in bronchiolar ROIs, there was a significant enrichment of gene sets including the interferon gamma response, interferon alpha response, TGF beta signaling, complement, apoptosis, hypoxia, reactive oxygen species pathway, and p53 pathway. Finally, in vascular ROIs, there was a significant enrichment of gene sets, including the inflammatory response, interferon gamma response, interferon alpha response, TNF-alpha signaling through NFkB, TGF beta signaling, IL2 STAT5 signaling, IL6 JAK STAT3 signaling, coagulation, complement, apoptosis, hypoxia, reactive oxygen species pathway, p53 pathway, and angiogenesis (Fig. 3g). These results indicate that the enriched gene pathways involved in the cytokine and cell damage responses against the virus vary depending on the type of the tissue structure. Notably, we found that the inflammatory response, as well as TNF-alpha and IL signaling pathways, were more pronounced in alveolar and vascular ROIs than in bronchiolar ROIs. Moreover, there was a significant upregulation of pathways related to coagulation and angiogenesis in vascular ROIs.

### Differential expression gene profiles involved in the cytokine response

**Fig. 4:**
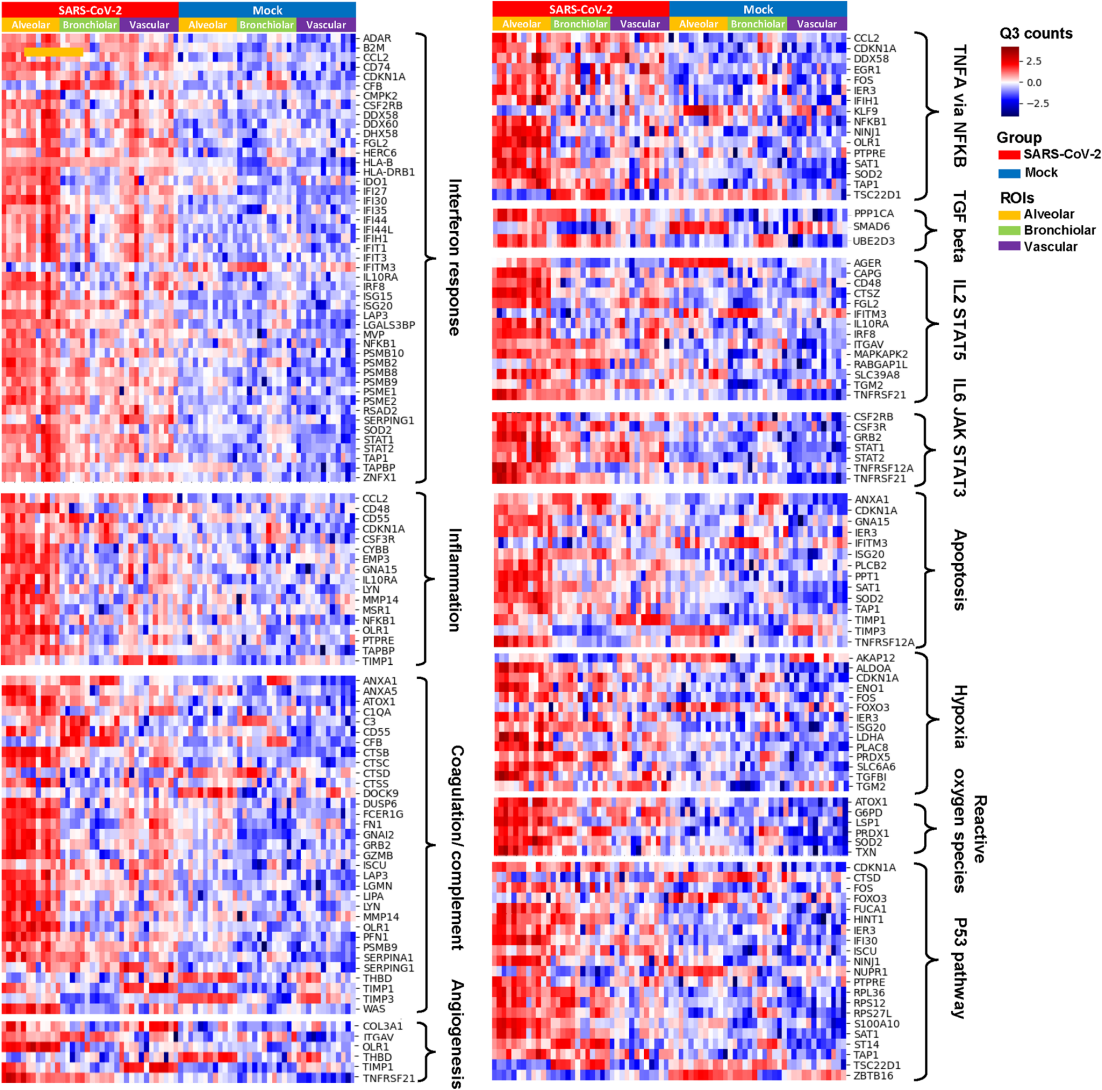
Heat maps shown for differentially expressed genes (DEGs) for the cytokine and cell damage response obtained by comparing Q3 normalized counts between SARS-CoV-2 and mock-infected lungs. Q3 normalized counts are colored in a blue-to-red gradient. Each group and microstructural ROI are represented with a different color.

Significant DEGs between SARS-CoV-2 and mock-infected ROIs were classified according to the functionally involved gene pathway in each structural ROI. Consistent with the results of GSEA, we found a significant co-upregulation of interferon response-related genes (*B2M*, *DDX60*, *PSMB8*, *PSMB9*, *PSME2*, and *STAT2*) in alveolar, bronchiolar, and vascular ROIs. Plus, *ADAR*, *CMPK2*, *DHX58*, *HERC6*, *IFI35*, *IFI44*, *IFIH1*, *IFIT3*, *ISG15*, *LAP3*, *PSMB9*, *PSME1*, *RSAD2*, and *TAP1* were significantly upregulated in alveolar ROIs. There were selective expression of genes involved in TNF and interleukin signaling pathways in alveolar and vascular ROIs. For DEGs involved in TNF-alpha signaling via NFkB, *CCL2*, *CDKN1A*, *DDX58*, *EGR1*, *FOS*, *IER3*, *IFIH1*, *NFKB1*, *NINJ1*, *SAT1*, and *SOD2* were upregulated only in alveolar and vascular ROIs. For DEGs involved in IL2–STAT5 signaling, *CTSZ* and *FGL2* were significantly upregulated in alveolar and vascular ROIs. Also, *CAPG*, *CD48*, *IL10RA*, *IRF8*, *ITGAV*, *MAPKAPK2*, and *SLC39A8* were significantly upregulated only in alveolar ROIs. For DEGs involved in IL6–JAK–STAT3 signaling, *CSF2RB*, *GRB2*, *STAT1*, *STAT2*, and *TNFRSF21* were significantly upregulated in alveolar and vascular ROIs. Also, *CSF3R* and *TNFRSF12A* were significantly upregulated only in alveolar ROIs (Fig. 4). These findings suggest that the lung in the early SARS-CoV-2 infection exhibits discrete regional transcriptional profiles associated with the cytokine response to the virus. Notably, our analysis identified gene signatures associated with the interferon response in all three structural ROIs, with TNF-alpha and IL signaling pathways being selectively enhanced in alveolar and vascular ROIs.

### Differential expression gene profiles involved in cell damage response

Consistent with GSEA results, there was a significant upregulation of inflammatory response-related genes (*TAPBP* and *TIMP1*) in alveolar and vascular ROIs. Plus, *CCL2*, *CD48*, *CDKN1A*, *CSF3R*, *CYBB*, *EMP3*, *GNA15*, *IL10RA*, *LYN*, *MMP14*, *MSR1*, *NFKB1*, *OLR1*, and *PTPRE* were significantly upregulated in alveolar ROIs. There were selective expression of genes involved in coagulation/complement pathways in alveolar and vascular ROIs. *CTSB*, *LGMN*, and *TIMP1* were significantly upregulated in alveolar and vascular ROIs. Also, *C1QA*, *C3*, *DUSP6*, *FN1*, *MMP14*, *OLR1*, and *SERPINA1* were significantly upregulated only in alveolar ROIs, whereas *CFB* and *SERPING1* were significantly upregulated only in vascular ROIs. For DEGs involved in angiogenesis, *COL3A1*, *TIMP1*, and *TNFRSF21* were upregulated in vascular ROIs. All three microstructural ROIs exhibited upregulation of cellular damage response, with high expression of genes enriched in apoptosis (*SAT1* and *TIMP1*), hypoxia (*ENO1*, *PLAC8*, and *TGFBI*), reactive oxygen species (*LSP1*, *PRDX1*, and *SOD2*), p53 (*FOS* and *IFI30*) pathways. Notably, these genes which indicate cellular damage, were predominantly expressed in alveolar ROIs (Fig. 4). These results indicate that DEGs involved in cellular damage response to virus vary depending on tissue microstructure. We found gene signatures associated with apoptosis, hypoxia, and reactive oxygen species, and p53 pathways in all three microstructural ROIs. Moreover, we found a selective upregulation of inflammatory response pathways in both alveolar and vascular ROIs.

### Microstructure-specific gene signatures in SARS-CoV-2 infected lungs

**Fig. 5:**
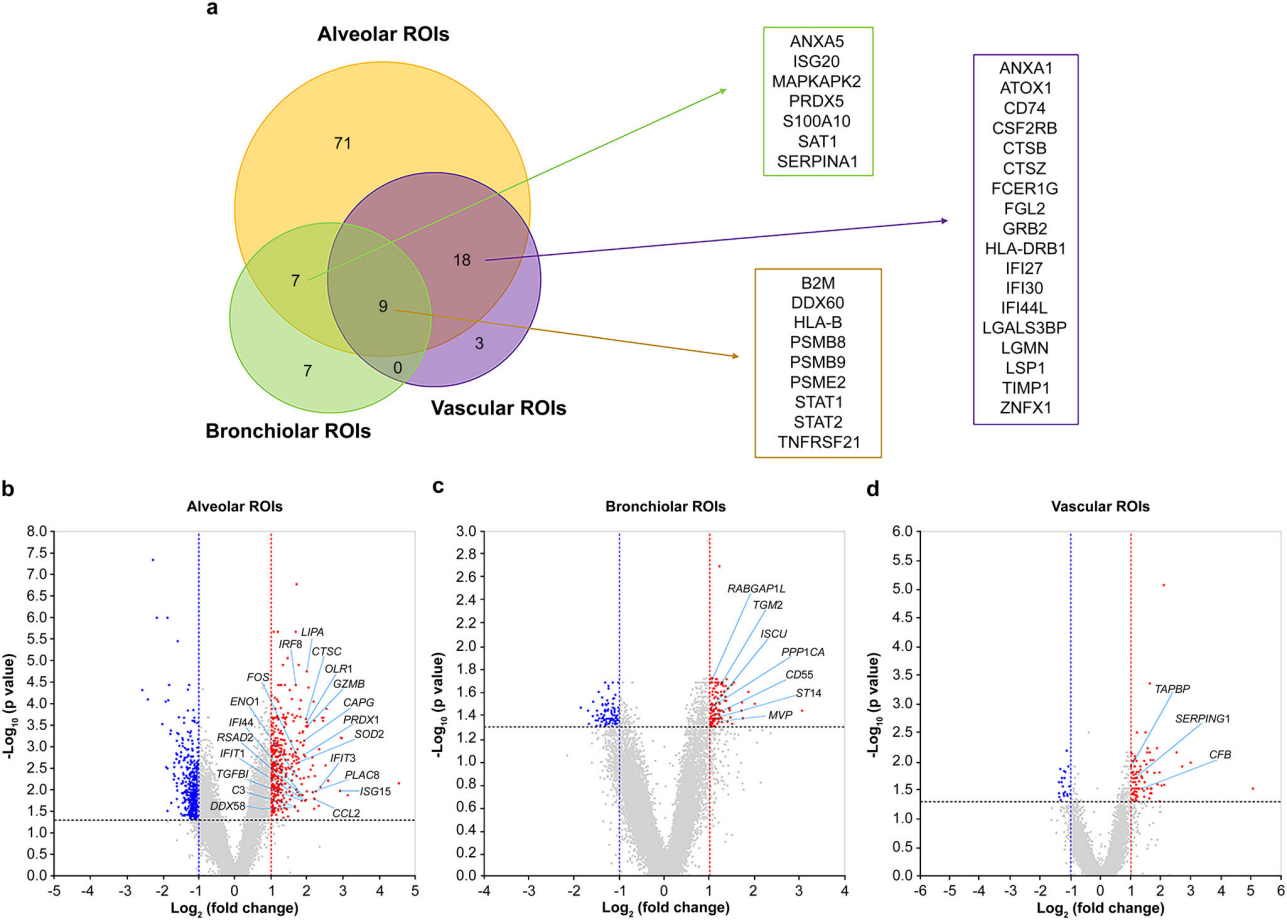
Pulmonary microstructure-specific COVID-19 gene signatures. **a** Venn diagram of microstructure-specific COVID-19 DEGs (versus mock, adjusted *P*-value < 0.05 and an absolute log2 fold-change >1). Volcano plot showing alveolar (**b**), bronchiolar (**c**), and vascular (**d**)-specific COVID-19 DEGs (versus mock, adjusted *P*-value < 0.05 and an absolute log2 fold-change >1).

We also developed a gene signature biomarker associated with each tissue microstructure. Regardless of the ROI of tissue structure, there was a consistent upregulation of genes including *B2M*, *DDX60*, *HLA-B*, *PSMB8*, *PSMB9*, *PSME2*, *STAT1*, *STAT2*, and *TNFRSF21* in COVID-19 lungs. Upregulation of genes, such as *ANXA5*, *ISG20*, *MAPKAPK2*, *PRDX5*, *S100A10*, *SAT1*, and *SERPINA1*, was evident in alveolar and bronchiolar ROIs. Also, the upregulation of genes, such as *ANXA1*, *ATOX1*, *CD74*, *CSF2RB*, *CTSB*, *CTSZ*, *FCER1G*, *FGL2*, *GRB2*, *HLA-DRB1*, *IFI27*, *IFI30*, *IFI44L*, *LGALS3BP*, *LGMN*, *LSP1*, *TIMP1*, and *ZNFX1*, was observed in alveolar and vascular ROIs. For alveolar ROIs, *C3*, *CAPG*, *CCL2*, *CTSC*, *DDX58*, *ENO1*, *FOS*, *GZMB*, *IFI44*, *IFIT1*, *IFIT3*, *IRF8*, *ISG15*, *LIPA*, *OLR1*, *PLAC8*, *PRDX1*, *RSAD2*, *SOD2*, and *TGFBI* were significantly upregulated (fold-change of >3 versus mock). For the bronchiolar ROIs, *CD55*, *ISCU*, *MVP*, *PPP1CA*, *RABGAP1L*, *ST14*, and *TGM2* were significantly upregulated (fold-change of >2 versus mock). For the vascular ROIs, *CFB*, *SERPING1*, and *TAPBP* were significantly upregulated (fold-change of >2 versus mock) (Fig. 5). These gene signatures observed in alveolar, bronchiolar, and vascular ROIs indicate the possibility of identifying microstructure-specific biomarkers for acute SARS-CoV-2 infections.

## Discussion

In the present study, we used the spatial transcriptome atlas analysis to identify pulmonary microstructure-specific COVID-19 gene signatures in the lungs of cynomolgus macaques experimentally infected with SARS-CoV-2. Conventional histopathology confirmed that this nonhuman primate model represents the phenotype of patients with severe COVID-19 based on the fact that lesions such as diffuse alveolar damage, bronchiolar inflammation, and vascular endotheliitis were clearly observed in this model. We performed spatial transcriptomic analysis of the major lung microstructures where these lesions occurred, including the alveoli, bronchioles, and blood vessels. The cytokine and cell damage responses were more robust in the alveolar and vascular regions than in the bronchiolar regions. Remarkably, we found significant differences in the expression of genes associated with the interferon pathway for antiviral activity, complement system, and inflammatory pathway mediating tissue damage in each microstructure. Furthermore, we found that clinically relevant biomarkers previously identified in blood or bronchoalveolar lavage fluid (BALF) samples collected from severe COVID-19 patients were selectively expressed in specific lung microstructures. In particular, several genes were proposed as biomarkers that are expressed universally, regardless of the type of microstructure. Moreover, we confirmed that COVID-19 signature genes such as *IFI27*—previously identified as a spatial transcriptional biomarker to differentiate between SARS-CoV-2 and influenza infections in human patients^14^ —were significantly upregulated in the lungs of a macaque acute infection model.

We found three patterns of pulmonary histopathological change in SARS-CoV-2-infected cynomolgus macaques partially consistent with COVID-19 patients^5^. However, a fibrotic pattern developing in patients with late stage of the disease was not observed in this acute infection macaque model. Notably, diffuse alveolar damage characterized by alveolar edema and hyaline membrane, was observed in the alveolar region. Previously, pathological changes in bronchiolar regions were underestimated; however, we observed clear patterns of bronchiolar epithelial degeneration and denudation with inflammatory cell infiltration. Furthermore, vascular changes, which are possibly a major driver of severe disease progression^4^, were observed with endothelialitis in cynomolgus macaques. These pathological findings were further confirmed by alterations in cell marker gene expression in the virus-infected lung. *AGER* and surfactant protein C (*SFTPC*), which are functional gene markers of type I and type II alveolar epithelial cells, respectively^15^, were downregulated in the virus-infected alveolar region. Moreover, the mucin gene *MUCL3* was downregulated in both alveolar and bronchiolar regions. The keratin genes *KRT14*, *KRT35*, and *KRT77* were downregulated in the alveolar region and *KRT35* was downregulated in the bronchiolar region. In contrast, the macrophage cell marker CD163 was significantly upregulated in the alveolar region and CD68 was upregulated in both alveolar and vascular regions. These results suggest a lower proportion of alveolar and bronchiolar lining cells as opposed to inflammatory cells in the lungs of cynomolgus macaques infected with SARS-CoV-2, which indicates a loss of normal tissue function. Although fibrotic changes were not prominent by H&E staining, several genes involved in collagen and protein attachment (e.g., *COL3A1*, *CTSA*, *CTSB*, and *CTSC*) were significantly upregulated in alveolar and vascular ROIs, and this finding is consistent with that of a previous macaque study^16^. This indicates early activation of fibrotic pathways in the infected lung parenchyma.

In the present study, the transcriptomes of the selected ROIs were distinguished according to the tissue microstructure and the presence of viral infection. The microstructure type of ROIs was the main biological factor demarcating the spatial transcriptome in the PCA plot. This may be attributed to varying predominant cell types in each microstructure, and is supported by previous studies distinguishing transcriptional profiles between tissues dominated by bronchiolar epithelial cells, alveolar macrophages, and type 2 pneumocytes^14^. Moreover, SARS-CoV-2 infection induced transcriptomic alterations in all three structural ROIs; however, some bronchial ROIs appeared to have a similar transcriptome regardless of infection, as observed in PCA. The presence of different patterns of tissue structures may be attributed to the subsequent GSEA results. A significant enrichment of the gene set involved in the cytokine response (Interferon, TNF-Alpha, TGF–BETA, IL2–STAT5, and IL6–JAK–STAT3) and cell damage response (inflammation, coagulation, complement, apoptosis, hypoxia, reactive oxygen species, and p53) were observed in both virus-infected alveolar and vascular ROIs compared with the corresponding mock-infected ROIs. However, there was no significant enrichment in several gene sets involved in the cytokine response (TNF-Alpha, IL2–STAT5, and IL6–JAK–STAT3) or cell damage response (inflammation and coagulation) in virus-infected bronchiolar ROIs. In patients who died due to COVID-19, upregulation of interferon- and cytokine-related gene pathways predominantly occurred in the large airway region^11^; however, the upregulation was more significant in the alveolar and vascular regions in macaques. This discrepancy can be attributed to the acute infection phenotype of the macaque model; therefore, it can be concluded that the host immune response to virus-induced cell damage during disease onset at the early stage of acute infection is robust in the alveolar and vascular regions rather than the bronchiolar region. Further investigation at different time points throughout the course of infection is needed to provide additional insights into the dynamic nature of the host response in distinct pulmonary microstructures. Additionally, only female animals were used in this study, so caution should be exercised when interpreting immune response profiles in the context of sex predilection. Greater T-cell activation in females has been reported in human patients, and T-cell responses and innate immune cytokines have been suggested to contribute to a differential disease progression by sex in patients with COVID-19^17^.

Consistent with PCA and GSEA results, heterogeneity according to lung microstructure was confirmed in the expression of genes related to cytokine response, particularly the interferon (IFN) response. Normally, the IFN response is required for protection against virus invasion, but its aberrant activation leads to the overproduction of proinflammatory cytokines, causing host tissue damage^18^. Hundreds of Interferon-stimulated genes (ISGs) are involved in the host IFN response, but their precise roles remain uncharacterized. The increased expression of ISGs was reported only in the lung samples with high SARS-CoV-2 during the early stages of infection, and this was associated with disease onset^19^. *ISG15*, which exaggerates the secretion of multiple proinflammatory cytokines and chemokines^18^, was significantly upregulated in alveolar ROI. *ISG20*, which exerts antiviral activities through RNase activity^20^, was upregulated in both alveolar and bronchiolar ROIs. *IFI27*, which may serve as an early transcriptomic biomarker in the blood samples obtained from COVID-19 patients^7,21^, was upregulated in alveolar and vascular regions, particularly with a significantly high fold-change in vascular ROIs. Interestingly, *RABGAP1L*, which enhances the IFN response to various RNA viruses and inhibits viral entry through endocytosis^22^, was highly expressed in the bronchiolar ROI. The results of our study support the local expression pattern of IFN response genes corresponding to virus distribution in the lung^10^ and further demonstrate that the upregulated IFN pathway genes differed based on the microstructure in the virus-infected area. The cause of this heterogeneity of IFN pathway gene expression across the lung microstructure during viral infection is unknown; however, this heterogeneity may contribute to the intrapulmonary nonuniform distribution of SARS-CoV-2.

Several genes associated with inflammation, which were previously considered biomarkers for severe COVID-19, were selectively upregulated based on pulmonary microstructure. Chemokine ligand 2 (CCL2), also referred to as monocyte chemoattractant protein 1 (MCP1), is involved in the recruitment of inflammatory monocytes or macrophages^23^, and S100A10 of the S100 calcium-binding protein family is involved in inflammation by increasing plasminogen production^24^. The elevated expression of genes encoding these two proteins was found in BALF and blood samples, respectively, obtained from patient with severe COVID-19^23,24^ and in the alveolar region of the lungs of SARS-CoV-2-infected macaques. These genes are enriched in pathways associated with activated macrophages which was further demonstrated by histopathological observations of intra-alveolar and interstitial macrophage infiltration in the lungs of macaques. The increase in tissue inhibitor of metalloproteinase 1 (TIMP-1), which is associated with the presence of highly activated neutrophils, was observed in the blood sample obtained from severe COVID-19 patients^25,26^. In our acute infection model, *TIMP1* gene was significantly upregulated in alveolar and vascular regions. This may be attributed to intra-alveolar and vascular neutrophil infiltration, which was observed in the early stage of the disease. Previously, oxidative stress was believed to contribute to mortality during SARS-CoV infection^27^. Oxidative stress-sensitive genes, such as *PRDX1* and *FOS*, were elevated in the blood of convalescent SARS-CoV patients^28^. Of these, peroxiredoxin 1 (PRDX1) is a potent scavenger of reactive oxygen species (ROS) and has been proposed as a potential therapeutic for SARS-CoV-2 infection^29^. These two genes were significantly upregulated in the alveolar ROI, suggesting that the regulation of the ROS pathway response to hypoxia is an important mechanism that can determine the threshold for irreversible parenchymal tissue damage during SARS-CoV-2 infection.

Common gene signatures were found in the alveolar, bronchiolar, and vascular regions of the lungs, with a focus on the cellular antiviral response and adaptive immunity. MHC class 1 genes, including *B2M* and *HLA-B*, which play a role in antigen presentation, cytotoxic T cell and NK cell activation, and host risk of infection^30,31^, were upregulated in alveolar, bronchiolar, and vascular ROIs. Moreover, *DDX60*—a recently discovered gene involved in the RIG-I-mediated type I interferon response and nuclease-mediated viral RNA degradation pathway^32^—was commonly upregulated. β-type proteasome subunits, *PSMB8* and *PSMB9*, which are involved in intracellular antigen processing^33^, were upregulated in all three structural ROIs. *STAT1* and *STAT2*, which mediate the type I and type III IFN response^34^, and *TNFRSF21*, a cell surface receptor gene that activates the JNK and NF-kB pathways^35^, were also upregulated in all three regions. These universal genes across the microstructures are key mediators of the host response to SARS-CoV-2; moreover, they partially overlap with the consistent increase in genes expressed in patients with COVID-19 regardless of the type of tissue^11^. Furthermore, these common signature genes are potential biomarkers for validating prognosis or direct targets for immune therapy. For example, the genes encoding proteasome subunits are overexpressed in COVID-19 patients during a hypoxic state and are associated with a decrease in lymphocyte count and positively correlated with inflammatory clinical markers^36^. Recent clinical trials have attempted to reduce the cytokine storm by regulating the JAK/STAT intracellular signaling pathway^37^.

As it is unclear whether the recently discovered biomarkers have a detrimental or protective role in specific microstructures, further studies are needed to investigate the role of each gene in SARS-CoV-2 pathogenesis. Moreover, because the gene profiles identified in the present study were based on spatial transcriptome data from regions containing both infected and uninfected cells, it is necessary to distinguish between cells with and without viral RNA via additional single cell RNA sequencing to discriminate between viral infection effects and bystander effects. In particular, because vascular regions without viral antigen also showed distinct lesions as described in a previous study^16^, the direct relationship between lesions and viral replication in endothelial cells requires further investigation. In addition, there were limitations regarding cross-reactivity between human WTA probe and macaque RNA targets, but we found that most probes had identity of >90% with their most closely matched targets (*n* = 16,201) and provided enough signal to be detected, as reported in previous studies on other species^38^. The final outcome of spatial transcriptome analysis is represented by the counts of liberated oligos, which helps non-pathologists quantify gene expression data. However, a histopathological background is required for selecting ROIs for analysis. Furthermore, while comparing the expression between different tissue samples, a careful consideration is required for normalization strategy to reduce batch effects^39^. Despite these limitations, spatial transcriptomics is a powerful tool that can complement bulk or single-cell RNA sequencing, where topological information is lost and limited to single-time analysis. Notably, this study approach involving the use of FFPE tissue is applicable not only to newly prepared specimens, but also to archival specimens for diagnostic purposes.

This is the spatial transcriptomic study of COVID-19 using NHPs to control the route and amount of virus infection. This study greatly reduced the heterogeneity observed in autopsy samples from human patients and enabled investigation of the host response to SARS-CoV-2 acute infection during the initial disease course. By elucidating the microstructure-specific host response, our findings provide further insight into the pathogenesis of COVID-19. Furthermore, we discovered novel biomarkers necessary for validating host risk factors and clinical prognoses following SARS-CoV-2 infection. Further studies of pulmonary microstructure-specific host responses to various respiratory pathogens and emerging SARS-CoV-2 variants are warranted.

## Methods

### Ethics statement

The authors confirm that the ethical policies of the journal, as noted on the author guidelines page, were adhered to and approved by the appropriate ethical review committee. The US National Research Council’s guidelines for the Care and Use of Laboratory Animals were followed. All animal procedures were approved by the KRIBB Institutional Animal Care and Use Committee (permit number KRIBB-AEC-20064).

### Animals and study design

Six female cynomolgus macaques (Macaca fascicularis) of Cambodian origin, between 3 and 6 years-old, were used for this study. Of these, three were infected with SARS-CoV-2, whereas the remaining three were included in the control group. The virus was inoculated into each macaque via multiple routes (intratracheal; 4 mL, oral; 5 mL, conjunctival; 0.5 mL, intranasal; 1 mL, and intravenous; 2 mL) at a concentration of 2.1 × 10^6^ 50% tissue culture infectious doses/mL ([TCID50]/mL) in a volume of 12.5 mL^40,41^. The control group was mock-infected with the same volume of PBS via the same routes of administration. Three days post-infection (dpi), all macaques were euthanized, necropsied; further, their respiratory organs were collected. These macaques were reared in indoor cages at the Biosecurity Level 3 (ABL-3) Animal Laboratory in the Korea National Primate Research Centre at the Korea Research Institute of Bioscience and Biotechnology (KRIBB).

### SARS-CoV-2

The SARS-CoV-2 virus (accession number NCCP43326) used in this study was provided by the National Culture Collection for Pathogens (Cheongju, Korea)^40,41^. The virus was isolated from a Korean patient and passaged three times in VERO cells before use. Further, the virus titer was determined in VERO cells using the Reed and Muench method and expressed as TCID50/mL.

### Histopathology

Prior to histopathological examination, macaque lungs were divided into six lobes: the right upper, right middle, right lower, left upper, left middle, and left lower. Tissue sections were prepared from a consolidated area of each lobe and stained with hematoxylin and eosin (H&E). If the consolidation area was not visually apparent, the same area identified in the other animals was sampled. Histopathological examination was performed using criteria modified from those used to determine histological patterns in clinical stages of COVID-19 patients^5^ (Table 1).

### Immunohistochemistry

Immunohistochemistry (IHC) was performed to confirm antigen distribution in the lung tissue sections from infected macaques. The IHC assay was carried out using a previously described method with slight modifications^40^. Briefly, 4-μm sections were deparaffinized and rehydrated with xylene and alcohol, and treated with 3% hydrogen peroxide to quench endogenous peroxidase. Heat-induced epitope retrieval was done using a pH 6 citric acid buffer. The tissue sections were blocked with 4% bovine serum albumin for 30 min at room temperature and incubated overnight at 4 °C with rabbit polyclonal antibodies against SARS-CoV-2 nucleocapsid (Sino Biological) diluted 1:1000. After two washes, the slides were incubated with enzyme-conjugated secondary antibody for 1 h at room temperature, visualized using 3,3-diaminobenzidine (DAB), and counterstained with hematoxylin.

### GeoMx digital spatial profiling (DSP) for whole transcriptome analysis (WTA)

NanoString GeoMx procedure described in a previous study was followed^42^. Formalin-fixed paraffin embedding (FFPE) tissue sections (4 μm) were prepared and immunofluorescence staining was performed to assess lung morphology using the epithelial cell marker PanCK (Novus, Clone AE1 + AE3), the immune cell marker CD45 (Novus, Clone 2B11 + PD7/26), and the macrophage marker CD68 (Santa Cruz, Clone KP1), along with Syto13 DNA nucleic acid staining. The tissue slides were hybridized with probes from the Human Whole Transcriptome Atlas, which targets more than 18,000 genes. BLAST measure of % Identity was used to estimate cross-reactivity between the probe and its closest matched target, which is tabulated in the Supplementary Data 1. Subsequently, they were loaded onto the GeoMx DSP. The selection of the region of interest (ROI) was guided by IHC staining of the virus nucleocapsid in serial sections. Oligos from the hybridized probes were released by photo-cleaving UV light (385 nm), which was projected onto the selected ROI and deposited into a 96-well plate. After drying overnight, the samples were resuspended in diethyl pyrocarbonate-treated water. Finally, sequencing libraries were constructed and subsequent sequencing and counting were carried out using the Illumina NovaSeq6000 following the manufacturer’s protocol.

### Limit of quantification (LOQ) and normalization strategy

LOQ was set to detect gene outliers. The limit was defined by the geometric mean of the negative probes multiplied by the square of geometric standard deviations of the negative probes, as generally recommended by the manufacturer. The data for each ROI passing LOQ filters were adjusted to achieve the same 75th percentile (Q3) of RNA expression signal using a normalization strategy generally used for whole transcriptome data analysis^9,11^. Briefly, this method involved estimating the Q3 for gene counts in each ROI, which were then normalized to the geometric mean of the Q3 for all ROIs. Further, normalization was conducted using all selected ROIs in the same process. The normalized counts were transformed into log_2_ values, which were used for subsequent analyses.

### Principal component analysis (PCA)

To identify significant trends and patterns in the gene expression datasets based on the experimental design, PCA was used to reduce the dimensionality of the data. This technique identifies the orthogonal dimensions of the dataset’s variability and simplifies the complexity of high-dimensional data by converting it into lower dimensions. The PCA ruled out individual (macaque) differences and experimental (GeoMX scan) batch variations resulting from the transcriptomic analysis. The NanoString R package was utilized for the analysis.

### Gene set enrichment analysis (GSEA)

The sequencing data for the virus- and mock-infected ROIs were analyzed using GSEA to identify significant associations with specific gene pathways. The GSEA 4.2.3 software package was utilized for the analysis along with the ‘HALLMARK’ gene set collection (consisting of 50 sets) obtained from the molecular signatures database (MSigDB version 7.1). A normalized enrichment score (NES) was calculated for each gene set and a false discovery rate (FDR) < 0.05 was considered significant enrichment. All analyses were conducted using a gene set permutation number of 1000.

### Differentially expressed genes (DEGs) analysis

DEGs between SARS-CoV-2-infected ROIs versus mock-infected ROIs were selected on the basis of statistically significant differences (Benjamini-Hochberg adjusted *P*-value < 0.05 and an absolute log_2_ fold-change >1).

### Reporting summary

Further information on research design is available in the Nature Portfolio Reporting Summary linked to this article.

### Supplementary information

Description of Additional Supplementary Files

Supplementary Data 1

Supplementary Data 2

Reporting summary

## Data Availability

The raw data of Spatial RNA-Sequencing are available on Sequence Read Archive (https://www.ncbi.nlm.nih.gov/sra; BioProject ID: PRJNA1002006). The source data behind the graphs in the paper is available in Supplementary Data 2. All other datasets used and analyzed in the current study are available from the corresponding author upon reasonable request.
