# Supplementary material for: Spatial transcriptome atlas reveals pulmonary microstructure-specific COVID-19 gene signatures in cynomolgus macaques
Source: Commun Biol. 2023 Aug 28;6:879. doi: 10.1038/s42003-023-05253-8 (PMC10462721; doi:10.1038/s42003-023-05253-8)
Supplement: Supplementary file 1 — Description of Additional Supplementary Files [file 42003_2023_5253_MOESM1_ESM.pdf]

Description of Additional Supplementary Files

File name: Supplementary Data 1
Description: BLAST measure of % Identity used to estimate cross-reactivity between human WTA probes and its closest matched target in macaque.

File name: Supplementary Data 2
Description: The source data behind the graphs in the paper.
